# Supplementary material for: Machine Learning Accelerates Crystallization for Structure Determination
Source: Angew Chem Int Ed Engl. 2026 May 4;65(25):e1218503. doi: 10.1002/anie.1218503 (PMC13266958; doi:10.1002/anie.1218503)

checkCIF/PLATON report

Structure factors have been supplied for datablock(s) lcz105

THIS REPORT IS FOR GUIDANCE ONLY. IF USED AS PART OF A REVIEW PROCEDURE FOR PUBLICATION, IT SHOULD NOT REPLACE THE EXPERTISE OF AN EXPERIENCED CRYSTALLOGRAPHIC REFEREE.

No syntax errors found. CIF dictionary Interpreting this report

Datablock: lcz105

Bond precision: C-C = 0.0144 A Wavelength=1.54184

Cell:	a=10.4806(2)	b=10.8443(3)	c=38.8707(6)
	alpha=96.016(2)	beta=95.956(1)	gamma=103.337(2)
Temperature:	100 K		

	Calculated	Reported
Volume	4237.49(16)	4237.49(16)
Space group	P -1	P -1
Hall group	-P 1	-P 1
Moiety formula	C33 H31 Ag3 F18 N6 O5	C15 H3 Ag3 F18 N6, C18 H28 O5
Sum formula	C33 H31 Ag3 F18 N6 O5	C33 H31 Ag3 F18 N6 O5
Mr	1257.25	1257.25
Dx,g cm-3	1.971	1.971
Z	4	4
Mu (mm-1)	12.187	12.187
F000	2456.0	2456.0
F000’	2467.28	
h,k,lmax	12,12,46	12,12,46
Nref	14977	14491
Tmin,Tmax	0.105,0.161	0.787,1.000
Tmin’	0.017	

Correction method= # Reported T Limits: Tmin=0.787 Tmax=1.000
AbsCorr = MULTI-SCAN

```
Data completeness= 0.968            Theta(max)= 66.600

R(reflections)= 0.0640( 10959)                          wR2(reflections)=
                                                        0.1832( 14491)
S = 1.048                 Npar= 1181
```

The following ALERTS were generated. Each ALERT has the format
test-name_ALERT_alert-type_alert-level.
Click on the hyperlinks for more details of the test.

Alert level A

```
PLAT971_ALERT_2_A Check Calcd Resid. Dens.  1.24Ang From N012          4.56 eA-3
```

Author Response: This Alert is due to presence of residual density in the presence of heavy metal atom (Ag).

```
PLAT971_ALERT_2_A Check Calcd Resid. Dens.  0.95Ang From N01E          4.14 eA-3
```

Author Response: This Alert is due to presence of residual density in the presence of heavy metal atom (Ag).

Alert level C

```
DIFMX02_ALERT_1_C  The maximum difference density is > 0.1*ZMAX*0.75
            The relevant atom site should be identified.
PLAT029_ALERT_3_C _diffrn_measured_fraction_theta_full value Low .      0.968 Why?
PLAT042_ALERT_1_C Calc. and Reported MoietyFormula Strings  Differ     Please Check
              Calc: C33 H31 Ag3 F18 N6 O5
              Rep.: C15 H3 Ag3 F18 N6, C18 H28 O5
PLAT094_ALERT_2_C Ratio of Maximum / Minimum Residual Density ....       3.39 Report
PLAT097_ALERT_2_C Large Reported Max.  (Positive) Residual Density       4.41 eA-3
PLAT213_ALERT_2_C Atom F020            has ADP max/min Ratio .....        3.3 prolat
PLAT213_ALERT_2_C Atom F02H            has ADP max/min Ratio .....        3.3 prolat
PLAT213_ALERT_2_C Atom F037            has ADP max/min Ratio .....        3.7 prolat
PLAT213_ALERT_2_C Atom F03M            has ADP max/min Ratio .....        3.1 prolat
PLAT213_ALERT_2_C Atom F01X            has ADP max/min Ratio .....        3.4 prolat
PLAT213_ALERT_2_C Atom F02O            has ADP max/min Ratio .....        3.1 prolat
PLAT213_ALERT_2_C Atom N011            has ADP max/min Ratio .....        3.5 prolat
PLAT220_ALERT_2_C NonSolvent   Resd 1  F   Ueq(max)/Ueq(min) Range        3.3 Ratio
PLAT230_ALERT_2_C Hirshfeld Test Diff for    N012     --C023     .        6.2 s.u.
PLAT230_ALERT_2_C Hirshfeld Test Diff for    N01E     --C02X     .        5.3 s.u.
PLAT234_ALERT_4_C Large Hirshfeld Difference F020     --C02V     .       0.19 Ang.
PLAT234_ALERT_4_C Large Hirshfeld Difference F02H     --C032     .       0.16 Ang.
PLAT234_ALERT_4_C Large Hirshfeld Difference F03M     --C02V     .       0.16 Ang.
PLAT234_ALERT_4_C Large Hirshfeld Difference F01G     --C03F     .       0.18 Ang.
PLAT234_ALERT_4_C Large Hirshfeld Difference F02O     --C03F     .       0.17 Ang.
PLAT234_ALERT_4_C Large Hirshfeld Difference F02P     --C03F     .       0.19 Ang.
PLAT234_ALERT_4_C Large Hirshfeld Difference N011     --C03B     .       0.17 Ang.
PLAT234_ALERT_4_C Large Hirshfeld Difference C02G     --C031     .       0.17 Ang.
```

```
PLAT342_ALERT_3_C Low Bond Precision on  C-C Bonds ..............    0.01445 Ang.
PLAT369_ALERT_2_C Long   C(sp2)-C(sp2) Bond  C029     - C02Z     .       1.53 Ang.
PLAT911_ALERT_3_C Missing FCF Refl Between Thmin & STh/L=    0.595        483 Report
               -1 11  0,   1-11  1,   1-11  2,   1-11  3,  -2-10  6,   8 -7  6,
                8 -7  7,   7  4  7,  -2-10  8,   7  4  8,  -2-10  9,  -4 -9  9,
                8  3 15,   8  3 16,  -9  0 22,   7 -4 26,   8 -8 27,   6 -6 27,
                3 -8 28,   8 -7 28,   6 -9 29,   7 -8 29,   8 -7 29,   8 -6 29,
                6 -5 29,   6 -1 29,   6 -9 30,   6 -8 30,   7 -8 30,   4 -7 30,
                   (  453 More Missing: see the .ckf listing file)
PLAT971_ALERT_2_C Check Calcd Resid. Dens.  1.30Ang From F02O           1.98 eA-3
```

Author Response: This Alert is due to presence of residual density in the presence of heavy metal atom (Ag).

```
PLAT971_ALERT_2_C Check Calcd Resid. Dens.  1.01Ang From F037           1.86 eA-3
```

Author Response: This Alert is due to presence of residual density in the presence of heavy metal atom (Ag).

```
PLAT971_ALERT_2_C Check Calcd Resid. Dens.  0.69Ang From N011           1.74 eA-3
```

Author Response: This Alert is due to presence of residual density in the presence of heavy metal atom (Ag).

```
PLAT971_ALERT_2_C Check Calcd Resid. Dens.  0.98Ang From F03M           1.58 eA-3
```

Author Response: This Alert is due to presence of residual density in the presence of heavy metal atom (Ag).

```
PLAT971_ALERT_2_C Check Calcd Resid. Dens.  1.06Ang From F03M           1.54 eA-3
```

Author Response: This Alert is due to presence of residual density in the presence of heavy metal atom (Ag).

```
PLAT973_ALERT_2_C Check Calcd Positive Resid. Density on      Ag01       1.28 eA-3
```

Alert level G

```
PLAT002_ALERT_2_G Number of Distance or Angle Restraints on AtSite          4 Note
PLAT003_ALERT_2_G Number of Uiso or U(i,j)  Restrained non-H-Atoms         18 Report
PLAT004_ALERT_5_G Polymeric Structure Found with Maximum Dimension          1 Info
PLAT007_ALERT_5_G Number of Unrefined Donor-H Atoms .............           1 Report
              H010
PLAT072_ALERT_2_G SHELXL First  Parameter in WGHT  Unusually Large       0.10 Report
PLAT083_ALERT_2_G SHELXL Second Parameter in WGHT  Unusually Large      19.44 Why ?
PLAT172_ALERT_4_G The CIF-Embedded .res File Contains DFIX Records          1 Report
PLAT173_ALERT_4_G The CIF-Embedded .res File Contains DANG Records          2 Report
PLAT177_ALERT_4_G The CIF-Embedded .res File Contains DELU Records          1 Report
PLAT178_ALERT_4_G The CIF-Embedded .res File Contains SIMU Records          3 Report
```

```
PLAT186_ALERT_4_G The CIF-Embedded .res File Contains ISOR Records          2 Report
PLAT188_ALERT_3_G A Non-default SIMU Restraint Value has been used     0.0100 Report
PLAT188_ALERT_3_G A Non-default SIMU Restraint Value has been used     0.0100 Report
PLAT188_ALERT_3_G A Non-default SIMU Restraint Value has been used     0.0100 Report
PLAT192_ALERT_3_G A Non-default DELU Restraint Value for SecondPar     0.0200 Report
PLAT242_ALERT_2_G Low    'MainMol' Ueq as Compared to Neighbors of       C01P Check
PLAT242_ALERT_2_G Low    'MainMol' Ueq as Compared to Neighbors of       C022 Check
PLAT242_ALERT_2_G Low    'MainMol' Ueq as Compared to Neighbors of       C02M Check
PLAT242_ALERT_2_G Low    'MainMol' Ueq as Compared to Neighbors of       C02T Check
PLAT242_ALERT_2_G Low    'MainMol' Ueq as Compared to Neighbors of       C02V Check
PLAT242_ALERT_2_G Low    'MainMol' Ueq as Compared to Neighbors of       C032 Check
PLAT242_ALERT_2_G Low    'MainMol' Ueq as Compared to Neighbors of       C025 Check
PLAT242_ALERT_2_G Low    'MainMol' Ueq as Compared to Neighbors of       C02C Check
PLAT242_ALERT_2_G Low    'MainMol' Ueq as Compared to Neighbors of       C02K Check
PLAT242_ALERT_2_G Low    'MainMol' Ueq as Compared to Neighbors of       C039 Check
PLAT242_ALERT_2_G Low    'MainMol' Ueq as Compared to Neighbors of       C03D Check
PLAT242_ALERT_2_G Low    'MainMol' Ueq as Compared to Neighbors of       C03F Check
PLAT333_ALERT_2_G Large Aver C6-Ring C-C Dist C01Z     -C02Q     .       1.44 Ang.
PLAT335_ALERT_2_G Check Large C6 Ring C-C Range C01Z     -C02Q           0.19 Ang.
PLAT720_ALERT_4_G Number of Unusual/Non-Standard Labels ..........        190 Note
              Ag01    Ag02    Ag03    Ag04    Ag05    Ag06    F008    F009
              O00A    F00B    F00C    O00D    O00E    H00E    F00F    O00G
              O00H    F00I    O00J    F00K    F00L    O00M    F00N    N00O
              N00P    N00Q    F00R    F00S    F00T    F00U    N00V    F00W
              N00X    F00Y    O00Z    O010    H010    N011    N012    F013
              F014    O015    N016    F017    F018    N019    F01A    N01B
              F01C    N01D    N01E    F01F    F01G    F01H    C01I    H01A
              H01B    H01C    C01J    F01K    C01L    C01M    C01N    C01O
              C01P    F01Q    C01R    C01S    C01T    H01T    F01U    C01V
              F01W    F01X    C01Y    H01D    H01E    C01Z    F020    C021
              H02A    H02B    C022    C023    C024    C025    C026    H026
              C027    H027    C028    C029    C02A    C02B    H02C    H02D
              C02C    C02D    H02S    C02E    H02T    C02F    C02G    H02Q
              H02R    F02H    C02I    H02U    C02J    C02K    C02L    H02E
              H02F    H02G    C02M    C02N    F02O    F02P    C02Q    C02R
              H02H    H02I    C02S    H02J    H02K    C02T    C02U    H02L
              H02M    C02V    C02W    C02X    C02Y    H02N    H02O    H02P
              C02Z    C030    C031    H03G    H03H    C032    C033    H03I
              H03J    H03K    C034    C035    H03L    H03M    H03N    C036
              H03O    H03P    F037    C038    H03A    H03B    C039    C03A
              H03C    H03D    C03B    C03C    H03E    H03F    C03D    C03E
              H03Q    H03R    C03F    C03G    H03S    H03T    C03H    H03U
              H03V    C03I    H03W    H03X    C03J    H03Y    Ha      C03L
              H03Z    Hb      F03M    C03N    H03     Hc
PLAT860_ALERT_3_G Number of Least-Squares Restraints .............        112 Note
PLAT909_ALERT_3_G Percentage of I>2sig(I) Data at Theta(Max) Still        57% Note
PLAT910_ALERT_3_G Missing FCF Reflection(s) Below Theta(Min)[Deg]=       3.46 Note
                0  0  1,   0  0  2,
PLAT941_ALERT_3_G Average HKL Measurement Multiplicity ..........         4.3 Low
PLAT969_ALERT_5_G The 'Henn et al.' R-Factor-gap value ..........       3.596 Note
              Predicted wR2: Based on SigI**2  5.10 or SHELX Weight 17.48
PLAT978_ALERT_2_G Number C-C Bonds with Positive Residual Density.          0 Info
```

---

2 **ALERT level A** = Most likely a serious problem - resolve or explain

0 **ALERT level B** = A potentially serious problem, consider carefully

32 **ALERT level C** = Check. Ensure it is not caused by an omission or oversight

```
 36 ALERT level G = General information/check it is not something unexpected

  2 ALERT type 1 CIF construction/syntax error, inconsistent or missing data
 40 ALERT type 2 Indicator that the structure model may be wrong or deficient
 11 ALERT type 3 Indicator that the structure quality may be low
 14 ALERT type 4 Improvement, methodology, query or suggestion
  3 ALERT type 5 Informative message, check
```

It is advisable to attempt to resolve as many as possible of the alerts in all categories. Often the minor alerts point to easily fixed oversights, errors and omissions in your CIF or refinement strategy, so attention to these fine details can be worthwhile. It is up to the individual to critically assess their own results and, if necessary, seek expert advice.

Validation response form

Please find below a validation response form (VRF) that can be filled in and pasted into your CIF.

```
# start Validation Reply Form
_vrf_DIFMX02_lcz105
;
PROBLEM: The maximum difference density is > 0.1*ZMAX*0.75
RESPONSE: ...
;
_vrf_PLAT029_lcz105
;
PROBLEM: _diffrn_measured_fraction_theta_full value Low .      0.968 Why?
RESPONSE: ...
;
_vrf_PLAT042_lcz105
;
PROBLEM: Calc. and Reported MoietyFormula Strings  Differ     Please Check
RESPONSE: ...
;
_vrf_PLAT094_lcz105
;
PROBLEM: Ratio of Maximum / Minimum Residual Density ....       3.39 Report
RESPONSE: ...
;
_vrf_PLAT097_lcz105
;
PROBLEM: Large Reported Max.  (Positive) Residual Density       4.41 eA-3
RESPONSE: ...
;
_vrf_PLAT213_lcz105
;
PROBLEM: Atom F020            has ADP max/min Ratio .....        3.3 prolat
RESPONSE: ...
;
_vrf_PLAT220_lcz105
;
PROBLEM: NonSolvent   Resd 1  F   Ueq(max)/Ueq(min) Range        3.3 Ratio
RESPONSE: ...
;
```

```
_vrf_PLAT230_lcz105
;
PROBLEM: Hirshfeld Test Diff for    N012     --C023     .        6.2 s.u.
RESPONSE: ...
;
_vrf_PLAT234_lcz105
;
PROBLEM: Large Hirshfeld Difference F020     --C02V     .       0.19 Ang.
RESPONSE: ...
;
_vrf_PLAT342_lcz105
;
PROBLEM: Low Bond Precision on  C-C Bonds ...............    0.01445 Ang.
RESPONSE: ...
;
_vrf_PLAT369_lcz105
;
PROBLEM: Long   C(sp2)-C(sp2) Bond  C029     - C02Z     .       1.53 Ang.
RESPONSE: ...
;
_vrf_PLAT911_lcz105
;
PROBLEM: Missing FCF Refl Between Thmin & STh/L=    0.595        483 Report
RESPONSE: ...
;
_vrf_PLAT973_lcz105
;
PROBLEM: Check Calcd Positive Resid. Density on      Ag01       1.28 eA-3
RESPONSE: ...
;
# end Validation Reply Form
```

PLATON version of 17/09/2025; check.def file version of 17/09/2025

---

duplicate check

No duplication found

Datablock lcz105 - ellipsoid plot

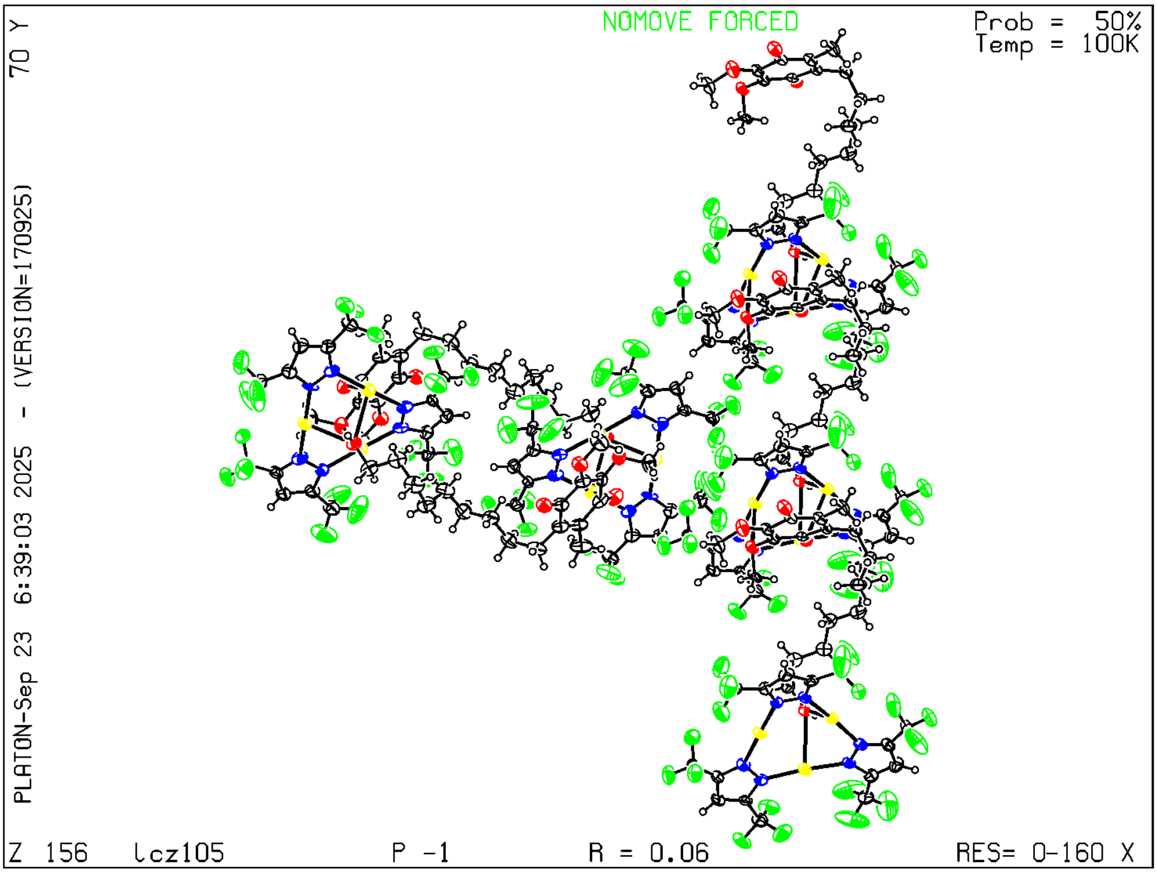

Supplement: Supplementary file 2 — Supporting File 2: anie72462‐sup‐0002‐DataFile.zip. [file ANIE-65-e1218503-s002.zip › supplementary_materials_crystallographic_data submit.zip!/cif_and_checkcif(1-114)/Ag3Pz3_105/Ag3Pz3_105_checkcif.pdf]
